# Retraction: Molecular Mechanism of Thiazolidinedione-Mediated Inhibitory Effects on Osteoclastogenesis

**DOI:** 10.1371/journal.pone.0229392

**Published:** 2020-02-13

**Authors:** The PLOS ONE Editors

After publication of this article [1], concerns were raised about similarities between the following panels in Figs 2, 3, and 7:

- Fig 2A, M-CSF, 10 ng/ml RANKL, 20 μM Ros (upper left quadrant) and M-CSF, 10 ng/ml RANKL, 20 μM Pio (lower right quadrant)
- Fig 2A, M-CSF, 10 ng/ml RANKL, 100 μM Ros and M-CSF, 10 ng/ml RANKL, 100 μM Pio
- Fig 2C, the left portion of the DMSO M-CSF panel for the Ros experiment (upper row) and the right portion of the–M-CSF Control 2 panel for the Pio experiment (lower row)
- Fig 3A, M-CSF, RANKL, 5 μM Ros (right) and M-CSF, RANKL, 5 μM Pio (left)
- Fig 7, middle and right panels are similar in panels A and C for 0h time point. The 20 μM Ros panel is similar to 20 μM Pio panel, 40 μM Ros panel is similar to 40 μM Pio panel.

The authors confirmed these issues and clarified that the original image data for experiments shown in Figs 2, 3, and 7 are no longer available. They provided the results of replication experiments conducted post-publication and several clarifications regarding the study design and methods of data analysis. The replication results were evaluated by a member of *PLOS ONE*’s Editorial Board who advised that when analyzed as per community standards the replication data do not support the results and conclusions reported in the article. The Academic Editor also raised concerns about the degree of concordance between quantitative results reported in the article for Rosiglitazone and Pioglitazone, for example in Fig 3.

The primary data underlying results in this article were not included with the published article although the Data Availability Statement for this article stated, “All relevant data are within the paper.” The authors commented that the images in Fig 1A, Fig 4A and Fig 4C in the original paper are primary data, and they noted that the raw data underlying Figs 1B, 4B, 4D, 5, 6, 8, and 9 are no longer available. As such the article does not comply with PLOS’ Data Availability policy.

In light of the above concerns, the *PLOS ONE* Editors retract this article.

The first author notified the journal that DZ and YW agreed with retraction. In addition, ZS and XF agreed with retraction. The other authors did not reply or could not be reached.
